# Effects of tailored quality improvement programme for effective medication management in high dependency in-patient psychiatry rehabilitation unit

**DOI:** 10.1192/bjo.2021.190

**Published:** 2021-06-18

**Authors:** Hina Tahseen, Jade Brown

**Affiliations:** Delfryn House, Cygnet Health Care

## Abstract

**Aims:**

To determine the effects of a tailored quality improvement programme for effective medication management including a reduction in prescription and administration errors in oral and depot psychotropic medication, patient education on medication and implementation of policies and guidelines.

**Background:**

Medication errors are common in hospital admissions and pose a threat to patient safety (Buckley et al. 2013). Medication errors may occur in different stages of the patient treatment process such as during prescribing, transcribing, preparing, dispensing, administration, and monitoring (Wang et al. 2015). In addition to these, for the detained mental health patients, the Mental Health Act 1983 legislation requires up-to-date treatment certificate compliance (Wales. Welsh Assembly 2008). A Quality Improvement programme to improve safe medication prescription and administration was designed for the patients admitted in Delfryn House, a mental health high dependency rehabilitation unit.

**Method:**

Using Plan-Do-Study-Act (PDSA) quality improvement methodology, a medication management committee was created under the leadership of Specialty doctor and Head of Care (HOC), and comprising of the consultant psychiatrists, specialty doctor, heads of care (ward managers), senior nurses, pharmacists, hospital manager and hospital director. The committee reviewed the medication errors reported in the last year and planned the Pre-Intervention Phase 1 and Post Intervention Phase II Audits.

The Intervention project was broadly divided into two domains---Doctors’ Prescription led by the Specialty doctor and the Nurses’ Medication Administration, led by the Head of care. Using the QI “theory of change” model, three primary drivers of “Safe Prescription and Administration”, “Patient Education” and “Policies and Guidelines Implementation” were established. The poster will have a demonstration of the complete drivers’ diagram.

Secondary drivers for “Safe prescription and administration” required inputs from doctors, nurses and pharmacists; Change ideas (Interventions) of introducing In-patient depot clinics, Daily 10-Points self-audit by clinic nurse, twice daily information about patients’ medication compliance in morning and evening electronic handovers, PDSAs with monthly audits of prescription and administration errors, monthly pharmacists’ audits for drug interactions and monitoring of adverse effects and rapid tranquilisations were implemented.

Secondary drivers and change ideas for “Patient Education” included discussions with Multidisciplinary teams, medication information leaflets being available to patients, discussion slots with pharmacists, self-administration of medication, and alternate self-management strategies instead of PRN medications.

Secondary drivers and change ideas for the “Policies and Guidelines Implementation” included steps to ensure all staff were aware of the policies for safe drug administration, rapid tranquilization and PRN utilisation, medication meetings minutes being circulated to all staff, and monthly audits for MHA1983 Section 57 treatment certificates for detained patients.

The medication Management Committee continued to meet on monthly basis to review the interventions, implementation of new strategies, and new recommendations on the basis of monthly mini-audits. A patient satisfaction survey on their knowledge about prescribed psychotropic medication was also conducted pre and post-intervention.

**Result:**

Results of Phase I and Phase 11 were compared. There was a significant reduction in prescription errors by doctors (19% to 3%) and medication administration (34% to 11%). Mental health documentation compliance improved from 77% to 98%. Patient satisfaction survey also demonstrated more knowledge about their prescribed psychotropic medication (15% to 32%). Two areas however did not show satisfactory improvements; There was not a significant improvement in acknowledgment or documentation of potential drug interactions or adverse events raised by pharmacists. Errors related to depot medication administration reduced in the initial two months, but increased again. The introduction of the Weekly Depot Clinic was not found successful by the administering nursing staff, and it was moved back to daily administrations.

**Conclusion:**

The formation of the medication management committee and the quality improvement programme showed significant improvement in most areas of effective medication management. The primary and secondary drivers with the change ideas gave structure to the intervention programme. The mini-audits using PDSA methodology helped to test different interventional strategies and to assess their impact and building upon the learning from previous results. This shows that for sustained effective medication management, this should not be a one-off exercise, and we need to continue learning and implementing newer strategies for continued effective medication, taking on-board the advice from MDT, nursing, patients, and carers.
